# Combined Antimicrobial Blue Light and Antibiotics as a Tool for Eradication of Multidrug-Resistant Isolates of *Pseudomonas aeruginosa* and *Staphylococcus aureus*: In Vitro and In Vivo Studies

**DOI:** 10.3390/antiox11091660

**Published:** 2022-08-26

**Authors:** Agata Woźniak, Mariusz Grinholc

**Affiliations:** Laboratory of Photobiology and Molecular Diagnostics, Intercollegiate Faculty of Biotechnology, University of Gdansk and Medical University of Gdansk, 80-307 Gdansk, Poland

**Keywords:** blue light, mouse model, photoinactivation, porphyrins, *Pseudomonas aeruginosa*, rose bengal, *Staphylococcus aureus*, synergy

## Abstract

Increased development of resistance to antibiotics among microorganisms promotes the evaluation of alternative approaches. Within this study, we examined the efficacy of antimicrobial blue light (aBL) with routinely used antibiotics against multidrug-resistant isolates of *Pseudomonas aeruginosa* and *Staphylococcus aureus* as combined alternative treatment. In vitro results of this study confirm that both *S. aureus* and *P. aeruginosa* can be sensitized to antibiotics, such as chloramphenicol, linezolid, fusidic acid or colistin, fosfomycin and ciprofloxacin, respectively. The assessment of increased ROS production upon aBL exposure and the changes in cell envelopes permeability were also goals that were completed within the current study. Moreover, the in vivo experiment revealed that, indeed, the synergy between aBL and antibiotic (chloramphenicol) occurs, and the results in the reduced bioluminescence signal of the *S. aureus* Xen31 strain used to infect the animal wounds. To conclude, we are the first to present the possible mechanism explaining the observed synergies among photoinactivation with blue light and antibiotics in the term of Gram-positive and Gram-negative representatives.

## 1. Introduction

Increased consumption and inappropriate applications of antibiotics in the medical sector and agricultural industry led to the development of multiple resistance mechanisms in microorganisms. Among these, the increased attention is nowadays directed toward two crucial pathogens: *Staphylococcus aureus* and *Pseudomonas aeruginosa*. These pathogens are responsible for hospital-acquired infections, especially in immunocompromised patients, due to their very high resistance level and the production of a broad spectrum of virulence factors [1]. Therefore, there is an urgent need to develop new therapeutic options to tackle drug resistance [2]. Alternative approaches against drug-resistant pathogens include bacteriophage therapy, antimicrobial peptides, lysins, antibodies or antimicrobial light therapy [3,4]. Light treatments involve the spectrum of visible light from 380 to 740 nm. Antimicrobial blue light (aBL) presented within the current study is one of the most attractive approaches. aBL refers to the light spectrum between 400 and 470 nm. It is an accepted hypothesis that application of this visible blue light leads to the excitation of the bacterial endogenous chromophores (e.g., flavins, iron-free porphyrins), which undergo the photochemical reaction [5,6]. Overall, this process results in the production of intracellular reactive oxygen species (ROS), which can cause lethal effects in bacterial cells as well as DNA cleavage, lipid and protein oxidation or cell membrane damage [7,8]. The overarching feature of aBL is that this method does not lead to the development of resistance in Gram-positive and Gram-negative bacteria, which has been thoroughly verified by our team [9,10]. aBL as a monotherapy is an effective tool for the eradication of pathogens and inactivation of their virulence factors [1,11]. Due to the non-specific mechanism of the action and the lack of resistance development, aBL may serve as an ideal component for combined treatments with other antimicrobial agents like antibiotics and lead to microbial resensitization to the action of routinely used antimicrobials [1,12].

Within the current study, we attempted to investigate which antimicrobial agents routinely used for *S. aureus* and *P. aeruginosa* treatment demonstrate the best activity with aBL revealing the synergistic effect. Moreover, the influence of endogenous chromophores was examined in the context of obtained synergy. Next, the study was aimed to investigate the safety of aBL toward eukaryotic cells and to assess ROS production and cell damage upon treatment (also in the presence of antibiotics). Finally, the last examined issue concerned the verification of observed synergy between aBL and antibiotics in in vivo experiments using a mouse model of infected wounds.

## 2. Materials and Methods

### 2.1. Characterisation of Clinical Isolates and Used Strains

In in vitro experiments, clinical *S. aureus* (4046/13, 1814/06) and *P. aeruginosa* (802, 805) strains isolated from blood samples were used. *S. aureus* were kindly provided by Joanna Empel from National Medicine Institute and *P. aeruginosa* by Nico T. Mutters from the Institute for Hygiene and Public Health at Bonn University Hospital. In assays investigating the influence of the porphyrin composition on the synergistic effect with aBL, two *S. aureus* strains were used: wild type (NCTC 8325-4) and its isogenic mutant (ΔhemB) with hemin biosynthesis gene interruption (hemB). Both strains (hemB and WT) were kindly provided by Karsten Becker from University Hospital Münster Institute of Medical Microbiology in Münster, Germany; moreover, construction of the mutant (hemB) was performed by C. von Eiff et al. [13]. In in vivo experiments, the bioluminescent strains, *S. aureus* Xen 31 and *P. aeruginosa* PAK were used.

### 2.2. Media and Culture Conditions

For all of the experiments, clinical isolates, wild-type strain NCTC 8325-4 and bioluminescent isolate Xen31 were cultivated at 37 °C in an orbital incubator for 16–10 h in Tryptic-Soy-Broth (TSB, Biomerieux, Craponne, France). For the cultivation of the ΔhemB mutant, erythromycin (ERY) at the final concentration of 2.5 μg/mL was added to the TSB broth and incubation was performed under the same conditions. The enumeration of bacterial colonies was performed on the solid plates containing the TSB medium with an addition of 1.5% agar (TSA) and ERY for hemB isolate.

### 2.3. Light Conditions

**Figure 1:**
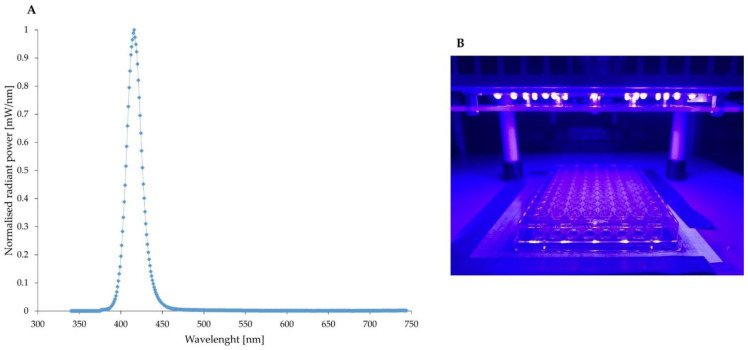
Normalised emission spectrum of light-emitting diode (LED) (**A**); Photograph of LED source used in experiments (**B**).

A light-emitting diode (LED) lamp (Figure 1A,B) manufactured by Cezos (Gdynia, Poland) and emitting 411 nm light (with an irradiance of 24 mW/cm^2^) was used in experiments. Irradiance measurements of the LED lamp were performed using the PM100D power/energy meter (Thorlabs, Ann Arbor, MI, USA).

### 2.4. Antibiotics

Chloramphenicol (CHL), Erythromycin (ERY), Ciprofloxacin (CIP), Doxycycline (DOX), Rifampicin (RIF), Fusidic acid (FA), Fosfomycin (FOF), Aztreonam (ATM), Clindamycin (CLI), Tigecycline (TGC), Imipenem (IPM), Colistin (CST), Gentamycin (GEN), Piperacillin (PIP), Tazobactam (TZB), Trimethoprim (TMP), Sulfamethoxazole (ST), Daptomycin (DAP), Linezolid (LZD), Oxacillin (OXA), Vancomycin (VAN), and Ceftazidime (CAZ) were purchased from Sigma (Darmstadt, Germany) and Cayman Chemicals (Ann Arbor, MI, USA). All stock solutions of 10 mg/mL were prepared in recommended solvents in non-transparent Eppendorf tubes and kept in −20 °C until use. 

### 2.5. Photoinactivation

Stationary growth-phase overnight cultures of clinical isolates were diluted to obtain the optical density of 0.5 McFarland in fresh TSB medium or PBS (Phosphate Buffered Saline), which corresponds to approx. 5 × 10^7^ colony-forming units per millilitre (CFU/mL). Then, for the experiments involving the antimicrobial blue light (aBL), the cells were immediately transferred to 96-well plates and irradiated with various doses of visible blue light. Afterwards, samples were diluted serially in PBS, seeded on agar plates (TSA) and incubated for 16–20 h at 37 °C in the incubator (Thermax, Dreieich, Germany). The enumeration of grown colonies was performed after 16–20 h of incubation, and the level of CFU/mL for each sample was estimated. Control samples without the addition of light were also involved in the experiment. All of the experiments were performed in three independent biological replicates.

### 2.6. Determination of Sub-Lethal Doses of Photoinactivation

According to our previous published data, the sub-lethal dose reduced bacterial viability by 0.5 to 2 log_10_ CFU/mL, and the lethal dose by more than 3 log_10_ CFU/mL.

### 2.7. Identification of the Minimal Inhibitory Concentrations (MIC) of Antibiotics and Lethal MIC Values for Photoinactivation Conditions

The Minimal Inhibitory Concentrations of tested antimicrobials were determined according to EUCAST (European Committee on Antimicrobial Susceptibility Testing) guidelines. Overnight cultures in a stationary phase-growth were prepared to obtain 0.5 McFarland suspension and diluted 10-fold in Mueller Hinton Broth (MHB) (Roth, Karlsruhe, Germany) to assess the number of cells approx. 5 × 10^6^ CFU/mL. In the next step, diluted cells were transferred to a 96-well plate with antibiotics to obtain the 2-fold range of agent concentrations ranging from 1024 to 0.00312 μg/mL. For the establishment of aBL lethality, diluted bacterial suspensions were transferred to the 96-well plate and immediately exposed to blue light. Afterwards, all plates were protected from evaporation with parafilm and incubated from 16–20 h at 37 °C in the incubator (Thermax, Dreieich, Germany). On the following day of the experiment, the turbidity of cell suspension was estimated, and the lowest concentration of the antimicrobial agent which inhibited the bacterial growth was defined as a MIC. The blue light dose that led to complete inhibition of bacterial growth was determined as a MIC of phototreatment. All the experiments were performed in three independent biological replicates.

### 2.8. Identification of the Interactions between Testes Phototherapies and Antibiotics—Recommended Methods for Synergy Testing

#### 2.8.1. Diffusion Assays

E-test strips containing the gradient concentration of antibiotics and disks containing one specific concentration of the antimicrobial agent were used in the diffusion methods (E-test and disk diffusion assay). Experiments were performed in accordance with the current guidelines for AST (antimicrobial susceptibility testing) recommendations provided by the EUCAST and presented within our previous publications [1,2,3]. To perform the experiment, overnight culture in a stationary growth phase was diluted to 0.5 McFarland in sterile PBS. For samples non-treated with photoinactivation (control), bacteria were spread with a cotton swab on the solid plates containing the Mueller Hinton Agar (MHA) (Sigma Aldrich, Darmstadt, Germany). For photoinactivation experiments with aBL, 1 mL diluted in PBS cells were transferred to a 12-well plate. Cells were then exposed to sub-lethal doses of aBL estimated in a PBS environment. After irradiation, phototreated cell suspension and control sample were spread on MHA plates, and after 15 min of incubation at room temperature (RT), discs and E-Tests were applied. An examination of diffusion experiments was performed after incubating plates at 37 °C in the incubator (Thermax, Dreieich, Germany) for 16–20 h. The inhibition zones were measured with the electronic caliper for disk diffusion assay, and the MIC values were determined. The synergistic effect was confirmed based on our previous published guidelines; thus, if changes in the zone of inhibition for photoinactivation-treated cells compared to the control samples are equal or more than 2 mm, then it confirms synergy. The difference in inhibition zone smaller than 4 mm indicates the antagonistic effect. For the E-test method, synergy is assessed when the MIC value of treated samples is 2-fold lower than the MIC value indicated for control samples. All the diffusion experiments were performed in three biological repetitions.

#### 2.8.2. Checkerboard Assay

This method involves the MIC values established for antibiotics and the aBL treatments. Briefly, bacterial cell suspensions were prepared the same as for the MIC establishment and then transferred into a 96-well plate. Antibiotics were added to the wells vertically (to obtain 2× MIC concentration) and then 2-fold dilutions of each tested compound were performed. After 15 min of incubation, plates were exposed to different doses of blue light (2× MIC, MIC, 1/2 MIC, 1/4 MIC and 1/8 MIC). After exposure to photoinactivation, plates were protected with parafilm and incubated at 37 °C in the incubator (Thermax, Dreieich, Germany) for 16–20 h. The next day, the bacterial growth assessment indicated if the synergistic or another effect (antagonism or indifference) effect occurred. The interpretation of the checkerboard result was based on the Fractional Index (FICI). (FICI = FIC_A_ + FIC_B_). FIC_A/B_ = MIC of factor A/B in combination/MIC of factor A/B alone. The synergistic effect is confirmed when FICI ≤ 0.5; antagonism was observed when FICI > 4; 4 < FICI > 0.5 means no interaction between tested factors.

#### 2.8.3. Postantibiotic Effect

This experiment was performed in accordance with our previous published studies; thus, the overnight culture of microorganisms was diluted in fresh TSB (1:20), and then the bacterial suspensions were mixed with MIC of antibiotic. All samples were then covered with aluminium foil and incubated for 2 h at 37 °C in an orbital shaker Innova40 (Brunswick, Hessen, Germany). Immediately after incubation, samples were centrifuged (3.5 min, 4500 rpm) and washed with a fresh TSB medium. After this step, cells were transferred in the amount of 100 μL to a 96-well plate and exposed to ½ MIC dose of blue light for aBL. In the next step, the optical density (λ 600 nm) of samples was measured for 15 h in multiplate reader Envision (PerkinElmer, Waltham, MA, USA) every 30 min. Obtained data were normalised and the postantibiotic effect (PAE) was determined based on the following equation: PAE = T − C (T, the time required to reach the optical density to value 0.5 (OD_600_) after removal of an agent; C, the time required to achieve the optical density (OD_600_) of untreated control samples). A postantibiotic effect value ≥ 3 h indicates synergy, whereas the 1.5 h ≤ PAE < 3 h confirms the partial synergistic effect.

### 2.9. Experiments Involving the Assessment of Mutagenic and Toxic Effects of aBL

Experiments concerning the determination of mutagenic and cytophototoxic effects of aBL were performed.

#### 2.9.1. Phototoxicity Assay

To perform this experiment, all procedures were performed in accordance with the protocol published by Michalska et al.; thus, HaCaT cells (CLS 300493, CLS Cell Lines Service GmbH, Baden-Württemberg, Germany) were grown in Dulbecco’s modified Eagle’s medium (DMEM) supplemented with 1 mM sodium pyruvate, 1 mM non-essential amino acids, 100 U/mL penicillin, 100 µg/mL streptomycin, 2 mM glutamine and 10% fetal bovine serum (all reagents were purchased from Life Technologies/Thermo Scientific, Darmstadt, Germany) [14]. The day before the experiment, cells were seeded in a 96-well plate in the number of 1 × 10^4^ cells/well in four repetitions for all tested conditions (photoinactivation and control). HaCaT cells were grown in a standard humified incubator (5% CO_2_) for 24 h and then exposed to various doses of aBL or the non-treated (control). After 24 h of irradiation, 10µL (12 mM) MTT reagent (1-(4,5-Dimethylthiazol-2-yl)-3,5-diphenylformazan) purchased from Sigma (Darmstadt, Germany) was added to each well and kept for 4 h at 37 °C in the incubator. In the next step of this assay, cells were lysed with DMSO, and the absorbance of formazan was established at 550 nm with plate reader Envision (PerkinElmer, Waltham, MA, USA).

#### 2.9.2. Mutagenicity Assay

This experiment was performed using the commercial kit Ames Penta 2 (Xenometrix, Allschwil, Switzerland). The day before the experiment, three independent biological cultures of each tested strain were prepared: *Escherichia coli* [uvrA], *Salmonella* Typhimurium [TA98, TA1535], 25 mL of growth medium. After 12–14 h incubation at 37 °C in the orbital shaker Innova40 (Brunswick, Hessen, Germany), cultures were diluted in an exposure medium and exposed to the various doses of aBL. Positive controls were also included in the experiment, thus, the 2-Nitrofluorene (for TA98 and 1535) and 4-Nitroquinoline-*N*-oxide (for uvrA) were added to the cultures to induce the mutations of the cells. Moreover, the negative control (without any treatment) was prepared. All of the cells, including negative control, were incubated after adding mutagen and/or aBL for 90 min in an orbital shaker at 37 °C Innova40 (Brunswick, Hessen, Germany). Afterwards, the exposure medium was added to the incubated cultures, and samples in the amount of 120 µL were partitioned into the 384 well plates (each sample was distributed to 48 wells separately in 3 technical repetitions). In the next step, all microplates were covered with sterile foil, placed in a plastic bag, and kept for 48 h at 37 °C in the incubator. The assessment of revertants was performed after 48 h. Thus, the number of grown colonies (in each well) was determined.

#### 2.9.3. Analysis of Eukaryotic Cell Growth Dynamic

To investigate the effect of visible blue light (aBL) on the growth rate of HaCaT (CLS 300493) cells, the day before the experiment, cells were seeded in the amount of 1 × 10^4^ cells/well in seven technical repetitions on E-plate PET plates (ACEA Biosciences Inc., San Diego, CA, USA) according to the protocol published by Michalska et al. [14]. Cells were cultured in the same conditions as described above in Section 2.9.1. and kept in the standard humified incubator with 5% CO_2_ for 24 h in the xCELLigence RTCA instrument (ACEA Biosciences Inc., San Diego, CA, USA). The next day, cells in the exponential growth rate (Cell index (CI) ≈ 2) were removed from the RTCA instrument, exposed to the various blue light doses and after the medium exchange, the plates were returned to the device. The CI was measured for each repetition every 10 min until the cells reached the plateau phase under tested conditions or if the cells did not survive post-irradiation.

### 2.10. Cell Permeabilisation

*S. aureus* isolate 4046/13 was cultivated in 25 mL of TSB medium for 4 h to obtain the logarithmic phase of growth, and then cells were centrifuged for 5 min/5000 rpm and resuspended in sterile PBS. Then, 1 mL of cells were exposed to aBL conditions in a 24-well plate and afterwards, SYTOX green was added to 100 μL of each sample to obtain a final concentration of 5 μM. To perform the cell membrane permeabilisation assay with propidium iodide (PI), the residual volume of the treated sample was mixed with PI to obtain a final concentration of 5 μg/mL according to the protocol published by Grinholc et al. [15]. The samples exposed to SYTOX green label were incubated for 15 min in the dark and the fluorescence, indicating the DNA leakage, was measured with multiplate reader Envision at 488/523 nm (excitation/emission wavelengths). Moreover, samples treated with PI were incubated in the dark for 30 min, then centrifuged and resuspended in a fresh portion of PBS in the amount of 200 μL. Immediately, the fluorescence signal was measured with Envision multiplate reader (PerkinElmer, Waltham, MA, USA) at 504/523 nm excitation and emission filters.

### 2.11. ROS Measurement

Hydroxyphenyl Fluoresceine; HPF (Thermofisher Scientific, Darmstadt, Germany) in the final concentration of 5μM was used with the cell suspension to assess the ROS production by endogenous chromophores, or in PBS with antibiotic—CHL was used to assess the increased ROS production by this agent upon aBL. All of the samples were incubated for 15 min in the dark and exposed to blue light doses. Immediately after exposure, the fluorescence signal was measured at (excitation/emission maxima) 490 nm/515 nm. Control samples containing the fluorescent probes but not exposed to visible light were also prepared. The experiment was performed in three technical and biological repetitions.

### 2.12. Investigation of the Porphyrin Composition Impact on the Synergistic Effect between aBL and Antibiotics

The overnight culture of the wild-type (WT) strain (NCTC 8325-4) was diluted in PBS and adjusted to the optical density of 0.5 MacFarland. Then, cells were transferred to a 96-well plate and exposed to various doses of aBL. Next, samples were serially diluted, spread on TSA plates, incubated overnight at 37 °C and afterwards, the sub-lethal dose of aBL was assessed. In the second part of the experiment, overnight cultures of the WT strain and ΔhemB mutant were in the amount of 1 mL exposed to the sub-lethal doses of aBL estimated for the WT. Irradiated samples were spread on MHA plates, incubated for 15 min in RT and the discs containing the antibiotic for susceptibility testing (the same as for the clinical isolates) were applied. In the next step, antibiograms were incubated overnight and the inhibition zones were measured. A similar experiment was performed for non-irradiated WT culture suspended in PBS in the amount of 0.5 McFarland. The differences in inhibition zones for wild type and the mutant lacking the possibility of heme synthesis were compared.

### 2.13. In Vivo Model of Mouse Wound Infected with Staphylococcus aureus/Pseudomonas aeruginosa—Verification of In Vitro Synergy

The 1st Local Ethical Committee for Animal Experiments in Krakow at the Institute of Pharmacology of the Polish Academy of Sciences (Warsaw, Poland) approved all experiments involving the procedures on animals. Twenty adult Balb/c mice aged 7–8 weeks were purchased from Charles River Laboratories (Wilmington, NC, USA). Animals were housed (five per cage) and maintained on a 12 h light-dark cycle with access to water and food ad libitum. The day before the experiment, mice were shaved on the dorsal surfaces, depilated with depilatory lotion, and the immunosuppressant—endoxan (150 mg/kg)—was injected intraperitoneal into each animal. The next day, overnight cultures of *S. aureus* (Xen31) or *P. aeruginosa* (PAK) cultured in a TSB medium were adjusted to 0.5 McFarland. Cells were centrifuged and resuspended in the physiological salt to obtain each 10 μL of culture 10^7^ CFU/mL. The wounds were created by making a 1 cm incision on the skin with a sterile needle, and immediately 10 μL of Xen31/PAK cells were applied to the damaged skin. Thirty minutes after infection of wound, mice were given: (i) antibiotic (1/2 MIC); (ii) aBL (MIC); (iii) antibiotic (1/2 MIC) + aBL (MIC). For experiments with Xen31 and PAK, chloramphenicol and piperacillin-tazobactam were used as antibiotics, respectively. The control group (iv) of mice were not given any treatment. Immediately after irradiation, the bioluminescence imaging of infected wounds was performed with the IVIS Spectrum imaging system (Caliper Life Sciences). During the bioluminescence, quantification mice were anaesthetised with isoflurane, and the luminescence was measured daily for up to 5 days. The quantification of the treatments was measured by the changes in bioluminescent signal, defined as an average radiance, and by observing the visual changes during the experiment.

### 2.14. Statistical Analysis

Statistical analysis was performed using the GraphPad Prism version 9.0 (GraphPad Software, San Diego, CA, USA) (https://www.graphpad.com/) (accessed on 19 August 2022). The statistical differences between groups were performed with one-way ANOVA with the significance level *p* < 0.05.

## 3. Results

### 3.1. Clinical Isolates of Pseudomonas aeruginosa and Staphylococcus aureus Respond Differentially to Photoinactivation in Various Environmental Conditions

**Figure 2:**
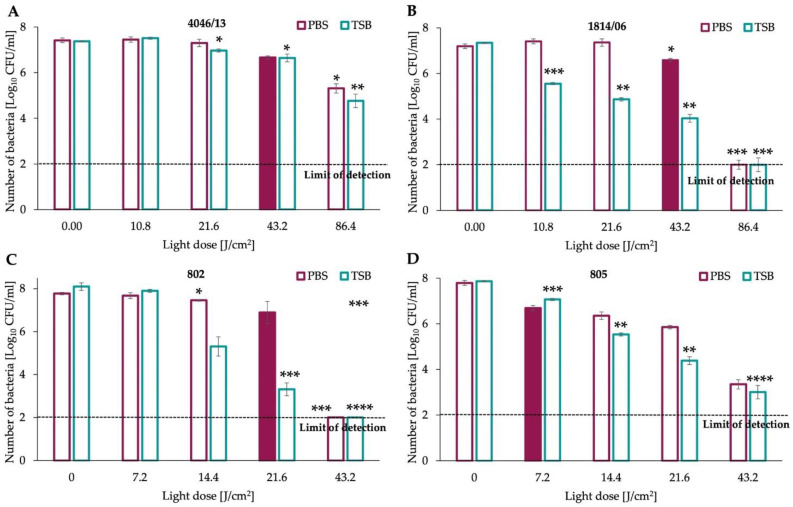
Antimicrobial blue light photoinactivation (aBL) of clinical isolates of *S. aureus:* (**A**) no. 4046/13; (**B**) no. 1814/06; (**C**) *P. aeruginosa* no. 802; (**D**) no. 805. Stationary growth-phase overnight cultures of clinical isolates were diluted to obtain the optical density of 5 × 10^7^ colony-forming units per millilitre (CFU/mL) in fresh TSB medium or PBS. Then, cells were transferred to 96-well plates and irradiated with various doses of visible blue light. Afterwards, samples were diluted serially in PBS, seeded on agar plates (TSA) and incubated for 16–20 h at 37 °C. The enumeration of grown colonies was performed after 16–20 h of incubation, and the level of CFU/mL for each sample was estimated. The experiment was performed in three independent biological replicates with 100 CFU/mL detection limit. Statistical significance (* *p* < 0.05; ** *p* < 0.01; *** *p* < 0.001; **** *p* < 0.0001) in comparison to samples not treated with aBL (0 J/cm^2^).

Photoinactivation of clinical isolates of Gram-positive representatives was more efficient in the TSB medium than in PBS (Figure 2A,B). Thus, the reduction in survival rate was comparatively lower when cells were distributed in PBS. The same observation can be drawn for Gram-negative isolates (Figure 2C,D) when exposed to various ranges of blue light in the TSB medium. Interestingly, isolates 802 and 805 respond better to photoinactivation than Gram-positive isolates 1814/06 and 4046/13 (lower doses of aBL were used to reduce the survival rate—up to 43.2 J/cm^2^ for isolates 802, 805). For *P. aeruginosa* isolate 802, the detection limit was reached after exposure to a blue light dose of 43.2 J/cm^2^ and a similar observation, but with no eradication to detection limit, can be made for the second isolate (805).

### 3.2. Sub-Lethal Doses of Photoinactivation in PBS Differs among Gram-Positive and Gram-Negative Species

Sub-lethal doses of aBL were demonstrated (based on the data presented in Figure 2A,B) as filled purple bars and these doses were implemented in the study involving the diffusion methods in the assessment of changes in resistance profile. For both isolates of *S. aureus,* the sub-lethal dose was evidenced as 43.2 J/cm^2^ and for *P. aeruginosa,* the aBL sub-lethal dose in PBS was estimated as 7.2 J/cm^2^ and 21.6 J/cm^2^, for strain no. 802 and 805, respectively (Figure 2C,D).

### 3.3. Examined Clinical Isolates Revealed XDR and MDR Categories of Resistance

The microdilution method was used to estimate the Minimal Inhibitory Concentrations (MIC) for tested isolates, thus a separate set of antimicrobials was used for Gram-negative and Gram-positive species. Table 1 represents MIC values that enabled the assignment of microorganisms to the resistance category according to the Magiorakos et al. [16]. Determination of susceptibility for each antimicrobial agent was performed with the clinical breakpoints published by the European Committee on Antimicrobial Susceptibility Testing (EUCAST) (https://www.eucast.org/clinical_breakpoints/ (accessed on 10 October 2021)). Based on the data published by the EUCAST, isolates 4046/13 and 1814/06 belong to the group of multidrug-resistant microorganisms (MDR) (Table 1). However, isolates 805 and 802 represent the XDR (extensively drug-resistance) profile of resistance (Table 1). Moreover, although the MIC parameter is mainly defined for antibiotics, for experimental purposes (checkerboard assay and postantibiotic effect), we also determined the alternative MIC values for phototherapy. Photoinactivation doses that lead to complete inhibition of bacterial growth were assigned as MIC for aBL (Table 1).

### 3.4. Recommended Methods for Synergy Testing Indicate the Increased Effectiveness of the Combination of aBL with Antibiotics for Clinical Isolates of S. aureus and P. aeruginosa

#### 3.4.1. Diffusion Methods Confirm Synergy after Pre-Treatment of Bacterial Cells with Blue Light

**Figure 3:**
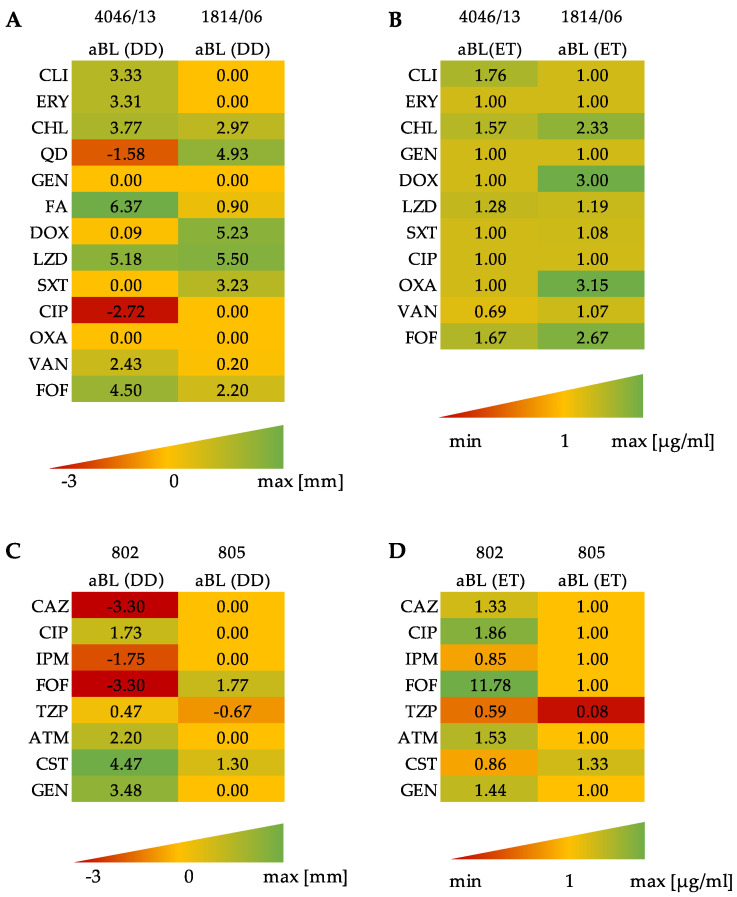
Graphical presentation of disk diffusion assay and E-test method. Figures present the differences in zones (in mm) of inhibition (in comparison to control) after exposure of isolates no. 4046/13 and 1814/06 to aBL (**A**); the ratio of control MIC values to MIC after exposure to aBL (**B**); (**C**,**D**) present the changes for isolates no. 802 and 805 from disk diffusion method and E-test, respectively, as it was present for *S. aureus* isolates. Each experiment was performed in three independent biological repetitions. Abbreviations: (DD) disk-diffusion; (ET) E-Test; (CLI) clindamycin; (ERY) erythromycin; (CHL) chloramphenicol; (QD) quinupristin-dalfopristin; (GEN) gentamycin; (FA) fusidic acid; (DOX) doxycycline; (LZD) linezolid; (SXT) trimethoprim-sulfamethoxazole; (CIP) ciprofloxacin; (OXA) oxacillin; (VAN) vancomycin; (FOF) fosfomycin; (CAZ) ceftazidime; (IPM) imipenem; (TZP) piperacillin-tazobactam; (ATM) aztreonam; (CST) colistin. MIN is defining the lowest relative MIC value from E-test; MAX defines the highest zone of inhibition (mm) and relative MIC value for the E-test.

E-test and disk diffusion assay presented in Figure 3A,B performed for clinical isolates of *S. aureus* (4046/13, 1814/06) indicate that the most pronounced synergy may be evidenced for CHL, LZD and FOF in the disk-diffusion method. Synergies with the E-test method were observed for the *S. aureus* isolate 1814/06 after exposure to aBL in the case of CHL, DOX, OXA and FOF (Figure 3B). On the other hand, a decrease in the susceptibility upon aBL treatment was indicated for CIP and Q-D. For Gram-negative isolates no. 802 and 805, it can be clearly seen that the isolate was more resistant to sensitization. The second isolate, no. 802, after exposure to aBL had a synergistic effect with ATM, GEN and CST confirmed with disk diffusion method and for FOF confirmed with E-Test (Figure 3C,D). For two antibiotics, CAZ and FOF, after exposure of isolate no. 802 to aBL, the decrease in the inhibition zones was identified.

#### 3.4.2. Simultaneous Blue Light Irradiation and Antibiotic Treatment Confirms Synergy for Multiple Antibiotics in Checkerboard Assay

Results from the checkerboard assay present the FIC_i_ index for the combined phototreatment of aBL and antibiotics. Table 2 presents the results obtained for two clinical isolates of *S. aureus*, and for isolate no. 1814/06, the synergy between aBL and antimicrobials was observed in the cases of CHL, FA, LZD, SXT and CIP. The second isolate, no. 4046/13, had synergy with aBL for CLI, CHL, FA, LZD and CIP (Table 2). Similarly to *S. aureus*, the synergy for clinical strains of *P. aeruginosa* were investigated with aBL and antibiotics (Table 3). Significantly less synergies were confirmed for *P. aeruginosa* isolates. In the case of strain no. 802, the synergy was confirmed for TZP, and for isolate no. 805, it was evidenced for CIP, CAZ, CST and FOF.

#### 3.4.3. Postantibiotic Effect Presents the Synergistic Effect for aBL and Antibiotics as a Significant Delay in Bacterial Growth of Tested Clinical Isolates

**Figure 4:**
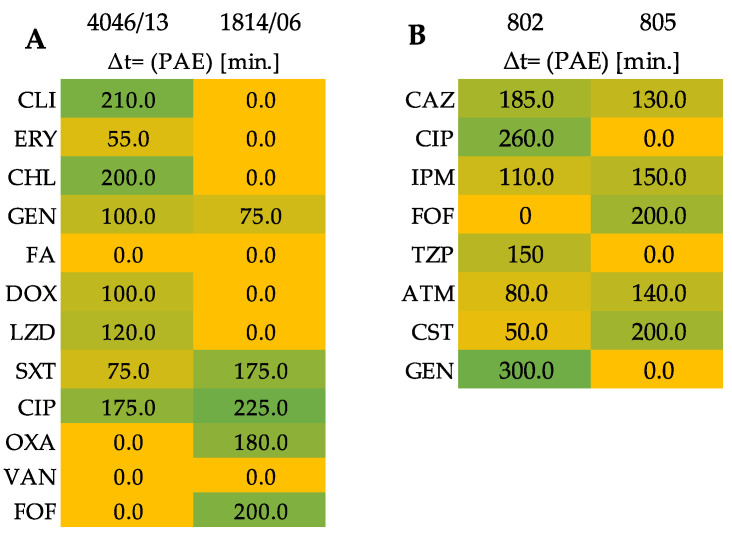
Graphical presentation of postantibiotic effect results (**A**) for isolate 4046/13 and 1814/06; (**B**) for isolate 802 and 805. Values of time below 90 min indicate the lack of synergy, whereas the unit of time between 90 min to 180 min confirms the partial synergy. All of the results above 180 min are recognized as a synergy.

**Figure 5:**
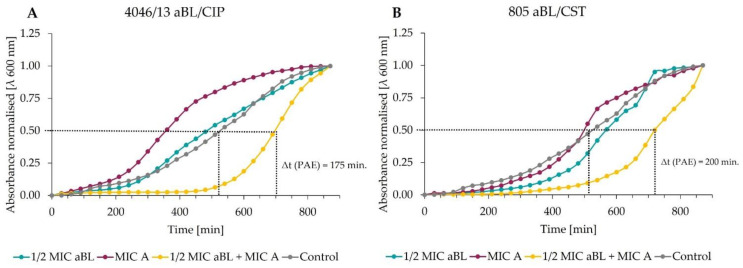
Postantibiotic effect growth curves. (**A**) Growth curve analysis of aBL/CIP combined treatment for *S. aureus* isolate 4046/13; (**B**) Growth curve analysis of aBL/CST combined treatment for the isolate of *P. aeruginosa* no. 805. Only one representative curve is presented. The overnight culture of microorganisms was diluted in fresh TSB (1:20), and then the bacterial suspensions were mixed with MIC of antibiotic. All samples were then covered with aluminium foil and incubated for 2 h at 37 °C. After incubation, samples were centrifuged and washed with a fresh TSB medium. Next, cells were transferred to a 96-well plate and exposed to ½ MIC dose of blue light. In the next step, the optical density (λ 600 nm) of samples was measured for 15 h every 30 min. Obtained data were normalised and the postantibiotic effect (PAE) was determined. Postantibiotic effect value ≥3 h indicates synergy, whereas the 1.5 h ≤ PAE < 3 h confirms the partial synergistic effect.

Assessment of the antimicrobial effect of the combined treatment (antibiotics and blue light) was established based on the growth curves for tested isolates. In the first step of the experiment, cells were exposed to an MIC dose of antibiotic which after 2 h incubation was removed. Then, the sub-lethal dose of aBL was applied. This method is different from those previously used due to the sequential application of the treatments, i.e., (1) antibiotic, (2) aBL. Figure 4 summarizes the results obtained for all studied strains and antimicrobials, and Figure 5 presents the synergistic effect via two representative curves, i.e., for ciprofloxacin obtained for *S. aureus* isolate no. 4046/13 and colistin obtained for *P. aeruginosa* isolate no. 805. The time required for reaching the growth point 0.5 OD_600_ for the combined treatment curve (1/2 MIC aBL + MIC A) was approx. 175 min in comparison to the control curve (Figure 4A). A similar time was required for *P. aeruginosa* isolate no. 805 to reach the OD_600_ value 0.5 for the growth curve 1/2 MIC aBL+ MIC A, when CST was used in the experiment (Figure 4B) in comparison to the control.

### 3.5. Antimicrobial Blue Light in Low Doses Did Not Cause the Mutagenic and Toxic Effect

**Figure 6:**
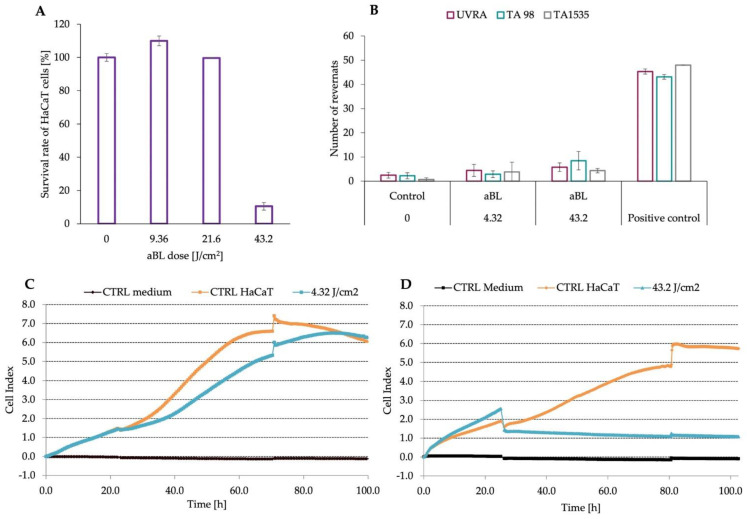
Assessment of phototoxicity, mutagenicity and influence on growth dynamic of eukaryotic cells upon aBL exposure to various light conditions. (**A**) Phototoxicity was assessed on the HaCaT cell line with the MTT assay; thus, eukaryotic cells were exposed to aBL doses and control–reference cells were included in the experiment. The day before the experiment, cells were seeded in a 96-well plate in the number of 1 × 10^4^ cells/well. After 24 h cultivation, the cells were exposed to various doses of aBL or non-treated (control). A total of 24 h postirradiation, 10µL (12 mM) MTT reagent (1-(4,5-Dimethylthiazol-2-yl)-3,5-diphenylformazan) was added to each well and kept for 4 h at 37 °C. Next, cells were lysed with DMSO, and the absorbance of formazan was established at 550 nm. (**B**) The mutagenic effect was assessed in Ames test with three different mutants (*E. coli* uvrA, *S.* Typhimurium TA 98 and TA1535). The assay included two blue light doses (4.32 and 43.2 J/cm2) and a positive and negative control (non-treated cells). The experiment was performed in three biological replicates, each replicating in three technical repetitions. *Escherichia coli* [uvrA] and *Salmonella* Typhimurium [TA98, TA1535] were diluted in an exposure medium and exposed to the various doses of aBL. Positive controls, i.e., the 2-Nitrofluorene (for TA98 and 1535) and 4-Nitroquinoline-N-oxide (for uvrA), were also included and added to the cultures to induce the mutations. The negative control (without any treatment) was also prepared. All of the cells were incubated after adding mutagen and/or aBL for 90 min at 37 °C. Afterwards, the exposure medium was added to the incubated cultures, and samples in the amount of 120 µL were partitioned into the 384 well plates (each sample was distributed to 48 wells separately in 3 technical repetitions). In the next step, all microplates were covered with sterile foil, placed in a plastic bag, and kept for 48 h at 37 °C. The assessment of revertants was performed after 48 h. Thus, the number of grown colonies (in each well) was determined. (**C**,**D**) The influence of the blue light on the growth dynamic of HaCaT cells was examined with two aBL doses (4.32 J/cm^2^ and 43.2 J/cm^2^), and the control cells (HaCaT CTRL) and medium control (CTRL medium) were included in the assay. The experiment was performed in 14 technical replicates. The day before the experiment, cells were seeded in the amount of 1 × 10^4^ cells/well on E-plate PET plates. Cells were cultured in the standard humified incubator with 5% CO_2_ for 24 h in the xCELLigence RTCA instrument. The next day, cells in the exponential growth rate (Cell index (CI) ≈ 2) were removed from the RTCA instrument, exposed to the various blue light doses and after the medium exchange, the plates were returned to the device. The CI was measured for each repetition every 10 min until the cells reached the plateau phase under tested conditions or if the cells did not survive post-irradiation.

Figure 6A presents the results of whether aBL can cause the phototoxic effect on eukaryotic cells. Therefore, we examined three aBL doses. The survival rate of HaCaT cells decreased by approx. 80% only for the highest dose of aBL (43.2 J/cm^2^). Two light doses, 9.4 J/cm^2^ and 21.6 J/cm^2^, were safe for eukaryotic cells (Figure 6A). We also attempted with the commercial Ames test to investigate the mutagenic effect of aBL, thus upon two implemented light doses, 4.32 J/cm^2^ and 43.2 J/cm^2^, we did not observe any increased number of revertants for the three tested mutants (Figure 6B). This observation is indicating that aBL is not a mutagenic treatment within the studied range of light doses.

Moreover, the last examination was focused on the analysis of the growth dynamics of HaCaT cells upon the aBL treatment. Figure 6C presents the growth dynamic upon exposure to a low dose of aBL, 4.32 J/cm^2^, and it evidences that the growth dynamic presented as the Cell Index is not significantly affected in comparison to control cells. However, an increased aBL dose (43.2 J/cm^2^) leads to a decrease in growth dynamic; thus, the CI decreased since the cells were exposed to blue light. Moreover, the cells did not reach the plateau phase; therefore, the applied aBL dose was lethal (Figure 6D).

### 3.6. aBL Leads to the Production of Various ROS and Increased Cell Permeability upon the Antibiotic Presence

**Figure 7:**
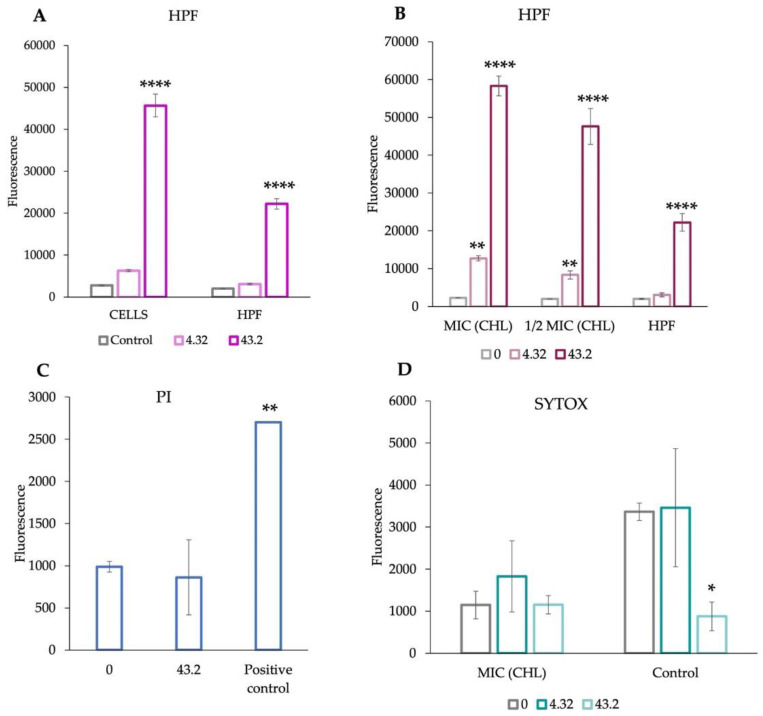
Assessment of the combination of CHL and/or aBL alone (in two doses) was performed with the implementation of (**A**) HPF probe (aBL); (**B**) HPF (aBL combined with CHL); (**C**) propidium iodide (aBL); (**D**) SYTOX green label (aBL combined with CHL); Each experiment was performed in three repetitions. All of the samples were incubated for 15 min in the dark and exposed to blue light doses. Immediately after exposure, the fluorescence signal was measured at (excitation/ emission maxima) 490 nm/515 nm. Control samples containing the fluorescent probes but not exposed to visible light were also prepared. Statistical significance (* *p* < 0.05; ** *p* < 0.01; **** *p* < 0.0001) in comparison to samples not treated with the aBL (0 J/cm^2^) or control.

To establish the ability of *S. aureus* cells to produce ROS, fluorescent probe HPF was used, and the autofluorescence of the probe was also taken into consideration during the experiment. Data present in Figure 7A clearly indicate that after exposure to aBL dose 43.2 J/cm^2^, the level of fluorescence increased for the cells compared to the value estimated for the HPF probe alone. This confirms that the production of ROS occurred upon aBL exposure even at the lower dose of aBL (4.32 J/cm^2^). Next, we aimed at the investigation of whether antibiotics upon aBL exposure can lead to increased ROS production. Therefore, the bacteria cells were not included in the experiment, despite the application of the HPF probe. As CHL was evidenced to give strong synergy using various synergy testing methods, this antimicrobial was chosen for ROS measurement and cell envelopes permeabilization studies. Data present in Figure 7B suggest that the CHL present in MIC and 1/2 MIC leads to the increased production of ROS upon two aBL doses in comparison to the HPF probe alone (control). On the other hand, we also evaluated whether the aBL can lead to increased cell permeabilization and, in consequence, increase the antibiotic influx into microbial cells. Figure 7C presents the data performed for the experiment with the involvement of propidium iodide, which plays a role as a cell membrane permeabilization indicator. Upon application of the blue light dose of 43.2 J/cm^2^, the permeabilization of the cells did not increase in comparison to the positive and negative control indicating no cell envelope permeabilization upon aBL. The last experiment was also focused on cell permeabilization; however, this was investigated in the presence of antibiotic (CHL). Data present in Figure 7D indicate that upon aBL treatment alone, even in the highest dose of 43.2 J/cm^2^, the DNA leakage of intracellular DNA was not observed, as the level of fluorescence of complex DNA-SYTOX was not higher in comparison to the control. No increase in the fluorescence signal from complex DNA-SYTOX was observed, which correlates with the PI experiment and indicates that no significant membrane permeabilization occurs upon aBL and aBL/CHL combined treatment.

### 3.7. Endogenous Porphyrins Present in Bacterial Cells Are Involved in the Synergistic Effect of aBL and Antibiotics in Staphylococcus aureus

There is a common hypothesis, that endogenous porphyrins and flavins present in bacterial cells are a crucial element in the mechanism of aBL. With application of the disc diffusion method, we investigated whether the isogenic mutant lacking heme production, and in consequence endogenous porphyrins production, can be sensitized to antibiotics as it is evidenced for the wild type (WT) strain. Table 4 presents the differences in the zone of inhibition for the WT and hemB isogenic mutant after exposure to a blue light dose of 64.8 J/cm^2^. This aBL treatment led to the reduction in bacterial viability for WT by 0.5–2 log_10_. For almost all antibiotics, the wild type *S. aureus* became sensitized after exposure to a sub-lethal aBL, with the exception of FOF. Contrary, in the case of the ΔhemB mutant no significant changes in zones of inhibition were observed, indicating the crucial role of endogenous porphyrins in the aBL mechanism which, in consequence, demonstrated synergies.

### 3.8. Synergistic Effect of aBL and CHL Rescues Mice from Wound Infection

**Figure 8:**
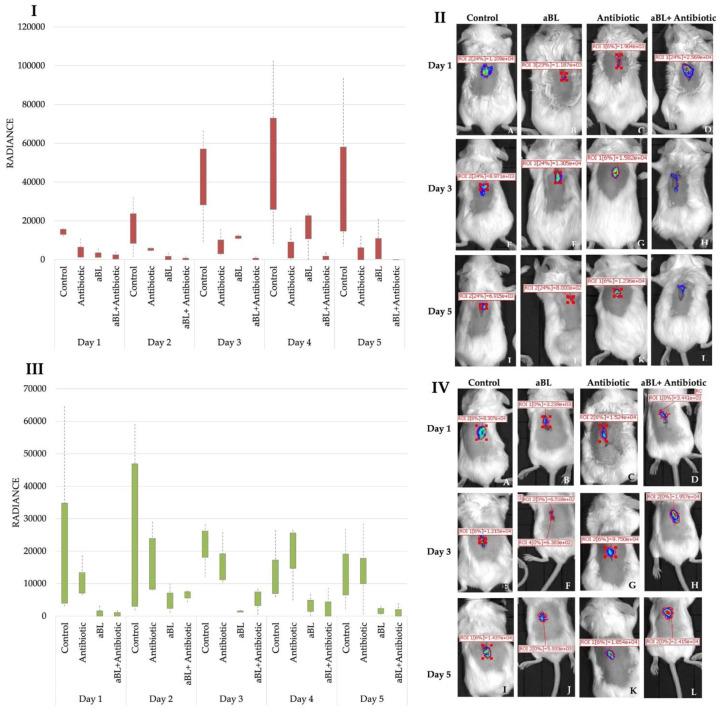
The combined therapy of wound infected with bioluminescent *S. aureus* Xen31 and *P. aeruginosa* PAK strains. Bioluminescence signals presented as radiance for all the experimental groups for wounds infected with Xen31 (**I**) and for wounds infected with PAK (**III**)**.** Bioluminescent images of infected wounds with Xen31 (**II**) and PAK (**IV**). The day before the experiment, mice were shaved on the dorsal surfaces, depilated with depilatory lotion, and the immunosuppressant—endoxan (150 mg/kg)—was injected intraperitoneal into each animal. The next day, overnight cultures of *S. aureus* (Xen31) or *P. aeruginosa* (PAK) were centrifuged and resuspended in the physiological salt to obtain each 10 μL of culture 10^7^ CFU/mL. The wounds were created by making a 1 cm incision on the skin with a sterile needle, and immediately 10 μL of Xen31/PAK cells were applied to the damaged skin. A total of 30 min after infection of wound, mice were given: (i) antibiotic (1/2 MIC); (ii) aBL (MIC); (iii) antibiotic (1/2 MIC) + aBL (MIC). For experiments with Xen31 and PAK, chloramphenicol and piperacillin-tazobactam were used as antibiotics, respectively. The control group (iv) of mice were not given any treatment. Immediately after irradiation, the bioluminescence imaging of infected wounds was performed with the IVIS Spectrum imaging system. The luminescence was measured daily for up to 5 days. The quantification of the treatments was measured by the changes in bioluminescent signal, defined as an average radiance, and by observing the visual changes during the experiment.

In vivo experiments were performed with the bioluminescent derivative of MRSA strain Xen31 and bioluminescent *P. aeruginosa* strain PAK. aBL was applied for wounds infected with Xen31 in a dosage of 8.6 J/cm^2^ and for wounds infected with PAK in a dose of 14.4 J/cm^2^. Both doses of aBL applied in in vivo experiments were the sub-MIC doses established for strain Xen31 and PAK, respectively. Moreover, based on the MTT experiment, both doses were identified as safe for eukaryotic cells (Data Not Shown). Antibiotics were used in MIC doses, for wounds infected with *S. aureus* CHL was used, and for *P. aeruginosa* TZP was applied. Though TZP was not evidenced with in vitro assays to give strong synergies for studied *P. aeruginosa* clinical isolates, it was chosen for in vivo studies as giving strong synergy for PAK isolate (Data Not Shown). The results presented in Figure 8I,II indicate that combined treatment led to the extinction of the infection in comparison to monotreatment (aBL or Antibiotic). Moreover, the decrease in the bioluminescent signal was observed even on day 3 of the experiment (Figure 8II). The application of monotherapies, i.e., aBL and Antibiotic (chloramphenicol), was also effective for wounds infected with Xen31; however, the best results were observed when these two therapeutic options were combined. On the other hand, wounds infected with bioluminescent PAK strain did not respond to combined aBL and antibiotic treatment. The infection in this group was not extinct even on the 5th day of the experiment. Monotreatments, i.e., aBL and antibiotic, were also ineffective in treatments of wounds (Figure 8III). Bioluminescent images of wounds infected with PAK presented in Figure 8IV indicate that the signals of bioluminescence were present in all the experimental groups till the end of the experiment (5th day).

## 4. Discussion

Photoinactivation as a monotreatment has been presented multiple times in literature as an efficient tool for the eradication of Gram-positive and Gram-negative microorganisms, viruses, parasites and fungi [17]. Moreover, the enhancement of biocidal action of fungicides, antibiotics or natural plant extracts against resistant-to-treatment organisms could be possible with the implementation of photoinactivation [12,18,19,20]. Presented within this study, two important pathogens, *Staphylococcus aureus* (SA) and *Pseudomonas aeruginosa* (PA), are nosocomial microorganisms responsible for approx. 1400 deaths in the USA due to pneumonia (PA) and more than 119,000 of deaths also in the USA were recorded for SA bloodstream infections [21,22]. Increased resistance to antibiotics forces the development of findings and solutions to fight with the lack of new antibiotics or to re-sensitize microorganisms to its action. Within this study, we investigate the possibility of using routine antibiotics against *P. aeruginosa* and *S. aureus* with antimicrobial blue light inactivation (aBL) in in vitro and in vivo studies.

Firstly, we investigated the effectiveness of aBL photoinactivation in two different environmental conditions: cells suspended in Phosphate Buffered Saline (PBS) and Tryptic Soy Broth (TSB) used for the cultivation of bacteria. *S. aureus* clinical isolates were less susceptible to aBL when diluted in PBS, and this observation was also drawn for *P. aeruginosa*; however, Gram-negative strains were more susceptible overall to aBL treatment. The differences in the effectiveness of photoinactivation depending on the culture medium were multiple times examined. For example, an experiment performed by dos Anjos et al. evidenced that the inactivation of various Gram-negative and Gram-positive microorganisms with blue light (λ 413 nm) was more effective in PBS in comparison to cells diluted in the suspension contaminated with milk [23]. In the current study, aBL efficacy was increased when using TSB medium instead of PBS. One must be aware that the photosensitizing compounds that play a crucial role in aBL are endogenous chromophores that could probably be washed out when TSB medium was replaced with PBS. It could lead to decreased concentration of PSs in an extracellular environment and result in decreased aBL effectiveness. Assessment of the sub-lethal and lethal doses for tested pathogens in these two conditions (TSB, PBS) was crucial due to the further implementations of obtained doses in the experiments concerning the evaluation of interactions between light and antimicrobials, therefore in Figure 1, only the sub-lethal doses of aBL were marked. Secondly, we characterized the antimicrobial resistance profile of clinical isolates, which confirmed that all of four isolates (4046/13; 1814/06; 802; 805) belong to the category of multidrug-resistant microorganisms, according to the outlines published by Magiorakos et al. [16].

According to the latest published review focused on combining the photoinactivation with antibiotics against these two pathogens, many of the published results are investigating the effectiveness of combined treatment in in vitro conditions only with a few antimicrobial agents. Within the current study, we examined the entire panel of antibiotics for each of the pathogen. The first method which allowed us to investigate the effectiveness of aBL with antibiotics was the disk diffusion method and E-Test. In both methods, microorganisms were at first exposed to aBL and afterwards, the antibiotics were applied as a disk or strip. *S. aureus* isolates had more synergistic effects than clinical isolates of *P. aeruginosa*; however, due to divergent peptidoglycan depth between these microorganisms and cell membrane structure, the differences in obtained results can be explained by these facts. The second method of synergy testing—checkerboard assay—also confirmed the success of combining aBL and antimicrobials, though light and antibiotics were applied simultaneously to bacterial cells. The results obtained for both *S. aureus* clinical isolates indicate that CHL, FA, LZD and CIP act synergistically with aBL. On the other hand, for *P. aeruginosa* clinical isolates, a lower number of synergies were confirmed. Single synergies were obtained for TZP, CIP, CST and FOF. The last method of synergy testing was based on the temporary treatment of cells with antibiotics in MIC concentration and subsequent treatment with aBL. The changes in the bacterial growth were the determinant of synergy for samples treated by combined treatment in comparison to the control groups in a postantibiotic effect method.

Table 5 presents all of the interactions detected within the current study for all combinations of aBL and antibiotics against two *S. aureus* clinical isolates. The green colour indicates the synergy. Moreover, the synergies that were already demonstrated in other published studies were marked as (+). A similar table was prepared for results obtained for clinical isolates of *P. aeruginosa* (Table 6). It is clearly visible that there is limited number of research concerning the aBL combination with antimicrobials. We as a first aimed to investigate the broad spectrum of antimicrobial agents against Gram-negative and Gram-positive representatives in combination with photoinactivation. One may see that aBL exhibits different potentiation effects for various antimicrobials representing various mode of action. We have demonstrated the synergies between aBL and antimicrobials targeted at protein as well as nucleic acids or cell envelopes synthesis (Table 5 and Table 6).

As mentioned above in literature, there are only a few articles which attempt to investigate the combination of aBL with antibiotics to eradicate ESKAPE representatives, *P. aeruginosa* and *S. aureus*. The experiment performed by Fila et al. proved that gentamycin, ceftazidime, or meropenem result in a synergistic effect against *P. aeruginosa* [1,28]. In contrast, another study indicates the lack of effectiveness of combining tigecycline, minocycline and aBL against this pathogen [29]. On the other hand, the combination of aBL and the antimicrobial agent was also examined for *S. aureus* within the experiments performed by Reznick et al., who demonstrated no effectiveness of aBL combined with minocycline and tigecycline. Up to this date, only two studies have confirmed the successful combination of ciprofloxacin and aBL against *S. aureus* [20]. Within our study, we observed various synergies for *S. aureus* and *P. aeruginosa* in different methods, therefore one may conclude that the order of the factors administration (blue light, antibiotics) may play an important role. The synergies observed, i.e., via disk diffusion method, differed from those obtained via checkerboard assay. Moreover, the investigation of the effectiveness of combined treatments was not evidenced in most of the mentioned above studies due to inappropriate methodology applied; therefore, we attempted to use the appropriate experimental protocol that was precisely described and critically analysed in our literature review [30]. The mechanisms of synergy between blue light and antibiotics are not revealed yet, however there are few potential explanations of this phenomenon. First of all, antibiotics upon exposure to the photons can undergo the photochemical reactions and play the role of producers of ROS. Experiments performed by He et al. imply that the presence of tetracyclines (e.g., demeclocycline) can potentiate the effect of photoinactivation with blue light resulting in the decrease in MIC value after aBL exposure [25]. Secondly, photoinactivation can lead to the inactivation of the enzymes or decreased expression of their coding genes that are responsible for the resistance to particular antibiotics. For example, the experiments performed by Boluki et al. evidenced the decreased level of expression of genes responsible for resistance to colistin in pan-drug-resistant strains upon photoinactivation [31]. However, this investigation was performed applying irradiation with red light (λ 630 nm) with the presence of exogenous PS, i.e., toluidine blue O. On the other hand, certain antibiotics (especially aminoglycoside, fluoroquinolones and β-lactam antibiotics) activate the tricarboxylic acid cycle, resulting in the metabolic changes which generate ROS [32]. Moreover, another possible explanation is linked with increased permeabilization and breakage of cell walls upon photoinactivation treatment, resulting in an increased antibiotic uptake and availability to cells [33]. Treatment with ROS-generating photoinactivation and subsequent treatment with the mentioned antibiotics may explain the synergy as an effect of the action of two ROS sources. Due to these facts, the action of antibiotics was potentiated upon aBL exposure (in checkerboard assay); after microbial culture exposure to aBL (diffusion methods); or after pre-treatment of bacterial culture with antibiotics in MIC concentration (in postantibiotic assay). It cannot be excluded that potentiation effect can also result from the mechanism of action of antibiotics.

An important issue discussed within the current work is the assessment of the toxicity of blue light in the context of in vivo experiments. The aBL doses implemented in animal experiments were lower than those used for clinical isolates in vitro. Despite this fact, aBL doses used in our investigations up to 21.6 J/cm^2^ did not influence the survival rate of HaCaT cell line. Implemented in our study, visible blue light is characterised by having a short wavelength (λ_max_ 411 nm) but the highest energy from the visible light spectrum [34]. The obtained results for the highest aBL dose (43.2 J/cm^2^) tested in MTT assay and used in the evaluation of cell growth dynamic with xCELLigence indicating significant cell inactivation are not surprising. Moreover, the research published by Liebman et al. also evidenced that blue light in higher fluences has a negative impact on human keratinocytes [35]. The experimental outcome from the Ames test excluded a mutagenic effect of aBL on the tested *E. coli* and *S.* Typhimurium mutants; however, it could not be excluded that the higher aBL doses may result in increased mutagenicity due to the cytotoxic effect of aBL in higher doses. Similar conclusions were drawn in research performed by Grinholc et al. who demonstrated the cytotoxic effect of New Methylene Blue and Toluidine Blue to *Salmonella* Typhimurium (TA98) upon treatment [36].

Investigation into the possible mechanism of aBL which is responsible for the synergistic interactions was another crucial part of the current study and it was investigated with the use of a Gram-positive representative. As a first, we confirmed that aBL leads to the production of various ROS with HPF probe in the presence of bacterial cells in irradiated suspension. The fluorescence level was higher after exposure of cells to aBL than the HPF probe (without cells) exposed to the same light conditions. Next, we confirmed that in the presence of CHL in MIC and 1/2 MIC, the ROS level increased also after exposure to aBL in two doses of light (4.32 J/cm^2^ and 43.2 J/cm^2^) when compared to the absence of antibiotic. This observation could explain the fact that in both *S. aureus* clinical isolates we observed synergistic effects for this antibiotic. In the case of cell envelopes permeabilization, propidium iodide and SYTOX green labelling were used. PI can easily uptake into the damaged bacterial cells, thus it may serve as a marker for membrane permeabilization upon aBL treatment. Upon the exposure of microbes to the highest aBL dose 43.2 J/cm^2^, the level of permeabilization was not higher when compared to the control. Applying the SYTOX green-fluorescent probe, which is able to bind to DNA released from damaged cells, did not result in the increased cell envelopes permeabilization upon aBL treatment nor when the highest aBL dose was administered. In other conditions, including antibiotic presence, the membrane permeabilization was not evidenced which correlates with the PI experiment. According to the literature, aBL (λ_max_ 405 nm) may lead to the *E. coli* cell permeabilization, however, this was not detected for *S. aureus* strain when SYTOX green was used [37].

Despite the investigation of ROS production via the aBL photoinactivation and its synergy with antimicrobials, we also took a closer look into the endogenous chromophores present in *S. aureus*. It is a first report that investigates whether the lack of porphyrin production can influence the synergies between light and antimicrobials. Therefore, we implemented the wild-type strain with the ability of porphyrins production and its isogenic mutant ΔhemB with impaired endogenous porphyrin production, for studies with aBL impact on their drug susceptibility profile using the disk diffusion method. We observed that inhibition zones for antimicrobials for mutant ΔhemB upon aBL exposure did not change in contrast to the wild-type strain. It clearly indicates that the endogenous porphyrins serve as an endogenous photosensitizing compound and play a crucial role for the aBL activity as well as its synergy with antimicrobials.

The culmination of in vitro research was the verification of the obtained results using in vivo models. With a mouse model of a wound infected with bioluminescent *S. aureus* strain Xen31, we confirmed that chloramphenicol with blue light is effective in wound healing in comparison to monotherapies alone (antibiotic, aBL). Furthermore, in the in vivo model, mice wounds infected with the Gram-negative PAK strain did not confirm the effectiveness of the combination of light and antimicrobials. The infection was not significantly inhibited up to the 5th day of the experiment. The obtained results could obviously be affected with the antimicrobial used for in vivo studies, i.e., piperacillin-tazobactam or the aBL dose. We expect that using other antimicrobial or increased aBL doses, the successful wound healing would be observed. Unfortunately, there is a lack of publications which confirm the effectiveness of aBL and antibiotics combined treatment with the use of in vivo models; thus, the current one is of high importance. Most of the in vivo studies are studying various photosensitizers, various visible light wavelengths or various agents which can potentiate the action of aBL as a monotherapy [7,38].

## 5. Conclusions

The results described in the current paper clearly demonstrate the enormous potential of this alternative treatment option resulting from combining blue light and antimicrobials. Obviously, further studies including the different blue light wavelengths, various light power and doses, as well as another microbial species or isolates representing different drug resistance profiles are required to support the accurate performance of in vivo studies, and to finally demonstrate the rationale for using this combined approach in the fight against drug-resistant pathogens.

## Figures and Tables

**Table 1:** Minimal inhibitory concentrations (MIC) of antibiotics and photoinactivation conditions.

Antibiotic Target	Antimicrobial Category	Antibiotic	*S. aureus*		*P. aeruginosa*	
			4046/13	1814/06	802	805
			MIC [µg/mL]			
**Protein synthesis (50S)**	Lincosamides	Clindamycin	0.25 (S)	0.01 (S)	ND	
	Macrolides	Erythromycin	256 ®	1024 (R)	ND	
	Phenicols	Chloramphenicol	64 (R)	128 (R)	ND	
	Streptogamins	Quinupristin-dalfopristin	ND			
**Protein synthesis (30S)**	Aminoglycosides	Gentamycin	1024 (R)	1024 (R)	16 (-)	1024 (-)
	Fucidanes	Fusidic acid	0.5 (S)	8 (R)	ND	
	Tetracyclines	Doxycycline	16 (R)	16 (R)	ND	
	Glycylcyclines	Tigecycline	2 (R)	16 (R)	ND	
**70S initiation complex**	Oxazolidinones	Linezolid	0.25 (S)	2 (S)	ND	
**Folic acid metabolism**	Folate pathway inhibitors	Trimethoprim-sulfamethoxazole	1024 (S)	16 (R)	ND	
**DNA-directed RNA polymerase**	Ansamycins	Rifampicin	0.03125 (S)	1024 (R)	ND	
**DNA gyrase**	Fluoroquinolones	Ciprofloxacin	32 (R)	32 (R)	2 (R)	128 (R)
**Cell-wall** **synthesis**	Anti-MRSA cephalosporins	Ceftaroline	ND			
	Carbapenems	Imipenem	ND		32 (R)	32 (R)
	Extended spectrum cephalosporins	Ceftazidime	ND		32 (R)	1024 (R)
	Antipseudomonal penicillins + β-lactamase inhibitor	Piperacillin-tazobactam	ND		64 (R)	1024 (R)
	Anti-staphylococcal β-lactams	Oxacillin	512 (-)	512 (-)	ND	
	Glycopeptides	Vancomycin	2 (S)	4 (R)	ND	
	Phosphonic acid	Fosfomycin (NR)	512 (R)	256 (R)	1024 (-)	1024 (-)
	Monobactam	Aztreonam	ND		32 (R)	32 (R)
**Cell membrane**	Lipopeptides	Daptomycin	64/32 (R)	32 (R)	ND	
	Polymyxins	Colistin	ND		1 (S)	1 (S)
			**Light dose [J/cm^2^]**			
**Phototherapy**	**aBL**	Blue light (411 nm)	86.4	86.4	21.6	15.8

Abbreviations: ND—not defined; R—resistant; S—susceptible, (-) category of resistance not defined according to clinical breakpoints.

**Table 2:** Checkerboard FIC_I_ calculation for *S. aureus* isolates.

Antibiotic	CLI	ERY	CHL	GEN	FA	DOX	LZD	SXT	CIP	OXA	VAN	FOF
1814/06	>0.5	>0.5	**0.312**	>0.5	**0.375**	>0.5	**0.5**	**0.25**	**0.5**	>0.5	>0.5	>0.5
4046/13	**0.437**	>0.5	**0.417**	>0.5	**0.4375**	>0.5	**0.5**	>0.5	**0.5**	>0.5	>0.5	>0.5

Bold font indicates possible synergistic interactions; (CLI) clindamycin; (ERY) erythromycin; (CHL) chloramphenicol; (GEN) gentamycin; (FA) fusidic acid; (DOX) doxycycline; (LZD) linezolid; (SXT) trimethoprim-sulfamethoxazole; (OXA) oxacillin; (VAN) vancomycin; (FOF) fosfomycin.

**Table 3:** Checkerboard FIC_I_ calculation for *P. aeruginosa* isolates.

Antibiotic	GEN	CIP	IPM	TZP	CAZ	ATM	CST	FOF
802	>0.5	>0.5	>0.5	**0.5**	>0.5	>0.5	>0.5	>0.5
805	>0.5	**0.5**	>0.5	>0.5	**0.5**	>0.5	**0.375**	**0.5**

Bold font indicates possible synergistic interactions; (GEN) gentamycin; (CIP) ciprofloxacin; (IMP) imipenem; (TZP) piperacillin-tazobactam; (CAZ) ceftazidime; (ATM) aztreonam; (CST) colistin; (FOF) fosfomycin.

**Table 4:** Changes in susceptibility profile of *S. aureus* WT (NCTC 8325-4) and isogenic mutant ΔhemB after aBL exposure.

Antibiotic	Control (WT)	aBL-64.8 J/cm^2^ (WT)	Control (ΔhemB)	aBL-64.8 J/cm^2^ (ΔhemB)
	[mm]	[mm]	[mm]	[mm]
CLI	26.6	**33.2**	6.0 *	6.0 *
ERY	27.6	**32.0**	6.0 *	6.0 *
CHL	30.5	**33.5**	31.2	30.5
Q-D	26.8	**30.3**	31.8	32.2
GEN	21.1	23.9	14.7	15.4
FA	33.4	**38.6**	33.2	34.3
DOX	31.8	**37.1**	34.7	34.5
LZD	30.6	**32.3**	33.3	32.1
SXT	26.0	**29.4**	20.7	21.0
CIP	22.7	**31.3**	32.2	28.4
OXA	27.4	**30.8**	19.8	20.6
VAN	11.9	13.1	12.7	12.5
FOF	49.2	33.4	30.1	31.0

* ΔhemB mutant is resistant to the ERY and CLI, thus the zones of inhibition even after the aBL exposure are equal to 6 mm. Abbreviations: (CLI) clindamycin; (ERY) erythromycin; (CHL) chloramphenicol; (QD) quinupristin-dalfopristin; (GEN) gentamycin; (FA) fusidic acid; (DOX) doxycycline; (LZD) linezolid; (SXT) trimethoprim-sulfamethoxazole; (CIP) ciprofloxacin; (OXA) oxacillin; (VAN) vancomycin; (FOF) fosfomycin. Bold font indicates a difference min. 2 mm in comparison to control, thus it confirms the synergy after exposure to aBL.

**Table 5:** Summarized results of synergy testing for *S. aureus* clinical isolates and synergies presented in a literature data for representative antimicrobial agents.

Antibiotic Target		4046/13				1814/06				Synergies Confirmed in Other Studies
		DD	E-T	CA	PAE	DD	E-T	CA	PAE	
Protein synthesis	CLI									
	ERY									
	CHL									
	QD									
	GEN									
	FA									
	DOX									(+) [24] (+) [25]
	LZD									(+) [26]
Nucleic acids	SXT									
	CIP									(+) [26] (+)[22]
Cell wall	OXA									(+) [27]
	VAN									
	FOF									

Abbreviations: (DD) disk-diffusion; (E-T) E-Test; (CA) checkerboard assay; (PAE) postantibiotic effect; (CLI) clindamycin; (ERY) erythromycin; (CHL) chloramphenicol; (QD) quinupristin-dalfopristin; (GEN) gentamycin; (FA) fusidic acid; (DOX) doxycycline; (LZD) linezolid; (SXT) trimethoprim-sulfamethoxazole; (CIP) ciprofloxacin; (OXA) oxacillin; (VAN) vancomycin; (FOF) fosfomycin. (+) indicate the positive effect of combination of aBL and antibiotic in other study. The green colour indicates the synergy; red colour indicates antagonism.

**Table 6:** Summarized results of synergy testing for *P. aeruginosa* clinical isolates and synergies presented in a literature data for representative antimicrobial agents.

Antibiotic Target		802				805				Synergies Confirmed in Other Studies
		DD	E-T	CA	PAE	DD	E-T	CA	PAE	
Protein synthesis	GEN									(+) [1]
Nucleic acids	CIP									
Cell wall/ Cell membrane	IPM									
	CAZ									(+) [1]
	TZP									
	FOF									
	ATM									
	CST									

Abbreviations: (DD) disk-diffusion; (E-T) E-Test; (CA) checkerboard assay; (PAE) postantibiotic effect; (GEN) gentamycin; (CIP) ciprofloxacin; (IMP) imipenem; (TZP) piperacillin-tazobactam; (CAZ) ceftazidime; (ATM) aztreonam; (CST) colistin; (FOF) fosfomycin. (+) indicate the positive effect of combination of aBL and antibiotic in other study. The green colour indicates the synergy; red colour indicates antagonism.

## Data Availability

The data presented in this study are available in the article.
